# Muir–Torre syndrome in Korean patients with sebaceous carcinomas: A retrospective study

**DOI:** 10.1016/j.jdin.2026.08.008

**Published:** 2026-09-02

**Authors:** Jinie Lee, Soomin Park, Ji Su Lee, Seong Jin Jo, Jee-Soo Lee, Moon-Woo Seong, Je-Ho Mun

**Affiliations:** a Seoul National University College of Medicine, Seoul, Republic of Korea; b Department of Dermatology, Seoul National University College of Medicine, Seoul, Republic of Korea; c Department of Dermatology, Seoul National University Hospital, Seoul National University College of Medicine, Seoul, Republic of Korea; d Department of Dermatology, Seoul Metropolitan Government – Seoul National University (SMG-SNU) Boramae Medical Center, Seoul, Republic of Korea; e Institute of Human-Environment Interface Biology, Seoul National University, Seoul, Republic of Korea; f Department of Laboratory Medicine, Seoul National University Hospital, Seoul National University College of Medicine, Seoul, Republic of Korea; g Cancer Research Institute, Seoul National University, Seoul, Republic of Korea

**Keywords:** DNA mismatch repair, epidemiology, genetic testing, Korea, Lynch syndrome, Muir-Torre syndrome, sebaceous adenocarcinoma, sebaceous gland neoplasm

*To the Editor:* Muir–Torre syndrome (MTS) is a rare autosomal dominant disorder caused by germline mutations in mismatch repair (MMR) genes and is characterized by the development of sebaceous neoplasms in patients with Lynch-associated visceral malignancies.^1^^,^^2^ Most epidemiologic data on MTS have been derived from Western populations, and its prevalence and clinical characteristics in East Asian populations remain poorly defined.^3^ Therefore, this study aimed to investigate the proportion and clinical profiles of Korean patients with sebaceous carcinoma (SC) meeting MTS criteria.

We retrospectively identified all patients with histologically confirmed SC diagnosed at Seoul National University Hospital between 2010 and 2025. This study was reviewed and deemed exempt (IRB No. E-2507-153-1659). Demographic variables, tumor characteristics, personal and family history, and the presence of Lynch-associated visceral malignancies were collected.^2^^,^^4^ MTS was defined as SC occurring in patients with either a history of Lynch-associated visceral malignancy (clinically defined MTS) or a pathogenic or likely pathogenic germline MMR gene variant (germline-confirmed MTS). *P*-value < .05 was considered statistically significant.

Ninety-one patients were diagnosed with 96 histopathologically confirmed SCs (Table I). Patients meeting MTS criteria did not differ significantly from those not meeting MTS in sex distribution, age at diagnosis, or number of tumors. However, extraocular involvement was significantly more common in MTS (58.3% [7/12] vs 22.8% [18/79]; *P* = .016). Among the 12 patients (13.2%) meeting the criteria for MTS, eleven (91.7%) had at least 1 visceral malignancy (Table II). One patient without a documented visceral malignancy had an extraocular SC, a family history of visceral cancers, and a novel pathogenic germline *mutL homolog 1* variant.

**Table I:** Patient demographics and characteristics of patients with sebaceous carcinoma according to Muir-Torre syndrome criteria

	Total (*N* = 91)	SC not meeting MTS criteria (*n* = 79)	SC meeting MTS criteria (*n* = 12)	*P*-value	OR (95% CI)
Demographics					
Sex				.756∗	
Male	39 (42.9%)	33 (41.8%)	6 (50.0%)		
Female	52 (57.1%)	46 (58.2%)	6 (50.0%)		
Age (y) at diagnosis	65.3 ± 13.2	65.6 ± 12.8	63.3 ± 16.1	.585†	
Ranges of age at diagnosis				.465∗	
≤39	2 (2.2%)	1 (1.3%)	1 (8.3%)		
40-49	11 (12.1%)	9 (11.4%)	2 (16.7%)		
50-59	16 (17.6%)	15 (19.0%)	1 (8.3%)		
60-69	22 (24.2%)	20 (25.3%)	2 (16.7%)		
70-79	31 (34.1%)	25 (31.6%)	6 (50.0%)		
80-89	7 (7.7%)	7 (8.9%)	0 (0.0%)		
90-99	2 (2.2%)	2 (2.5%)	0 (0.0%)		
Tumor characteristics					
Number of SCs per patient, mean ± SD	1.05 ± 0.23	1.04 ± 0.19	1.17 ± 0.39	.133‡	
Patients with ≥2 SCs	5 (5.5%)	3 (3.8%)	2 (16.7%)	.128∗	
Patients according to tumor location				**.016**	**4.74 (1.34-16.77)**
Periocular	66 (72.5%)	61 (77.2%)	5 (41.7%)		
Extraocular	25 (27.5%)	18 (22.8%)	7 (58.3%)		

For categorical variables, data are presented as *N* (%), and for continuous variables, as mean ± SD.

Bold font indicates statistical significance (*P* < .05).

*CI*, Confidence interval; *MTS*, Muir-Torre syndrome; *OR*, odds ratio; *SC*, sebaceous carcinoma; *SD*, standard deviation.

∗ Fisher’s exact test.

† Independent samples *t* test.

‡ Mann–Whitney *U* test.

**Table II:** Characteristics of patients meeting Muir-Torre syndrome criteria

Case	Sex/age at SC diagnosis (y)	SC location (periocular vs extraocular)	Visceral malignancy (age at diagnosis, y)	MMR IHC (result)	Germline variants of MMR genes	ACMG/AMP classification
1	F/31, 41∗	Left upper lip (extraocular) Posterior scalp (extraocular)	-	MLH1 (loss) MSH2/MSH6/PMS2 (retained)	MLH1: c.303_304dup (p.Glu102Valfs∗7)	Pathogenic
2	M/42	Right upper eyelid (periocular)	Colorectal (40)	-	-	-
3	M/47	Nasal tip (extraocular)	Colorectal (32) Colorectal (32) Gastric (38)	-	-	-
4	M/55	Vertex of scalp (extraocular)	Urothelial (49)	-	-	-
5	F/64	Left cheek (extraocular)	Colorectal (61) Colorectal (61)	MSH2/MSH6 (loss) MLH1/PMS2 (retained)	MSH2: c.1014_1021delinsGTTTACTGG (p.Gln339Phefs∗19)	Likely pathogenic
6	F/65	Left cheek (extraocular)	Colorectal (60)	MLH1/MSH2 (retained)	-	-
7	M/72	Left lower eyelid (periocular)	Prostate (78)	-	-	-
8	M/73	Neck (extraocular)	Colorectal (60)	-	-	-
9	F/76	Right lower eyelid (periocular)	Gallbladder (74)	-	-	-
10	M/78	Left upper eyelid (periocular)	Colorectal (50)	-	-	-
11	F/78	Left upper eyelid (periocular)	Gastric (75)	-	-	-
12	F/79†	Right cheek (extraocular)	Urothelial (60) Colorectal (66)	MSH2/MSH6 (loss) MLH1/PMS2 (retained)	MSH2: c.310A>T (p.Lys104∗)	Likely pathogenic

MTS classification: Case 1, germline-confirmed; Cases 2-4 and 6-11, clinically defined; Cases 5 and 12, clinically defined and germline-confirmed.

*AMCG*, American College of Medical Genetics and Genomics; *AMP*, Association for Molecular Pathology; *IHC*, immunohistochemistry; *MLH1*, mutL homolog 1; *MMR*, mismatch repair; *MSH2*, mutS homolog 2; *MSH6*, mutS homolog 6; *SC*, sebaceous carcinoma; *SCC*, squamous cell carcinoma.

∗ This patient developed 2 SCs at 31 and 41 years of age.

† This patient also had squamous cell carcinoma on the forehead at 74 years of age and keratoacanthoma on the right cheek at 79 years of age.

Various visceral malignancies were identified in MTS (Table II). Colorectal adenocarcinoma was the most common (60%). In most cases, visceral malignancies preceded the diagnosis of SC. MMR immunohistochemistry and germline genetic testing results were available for 4 and 3 patients, respectively. SC tissue demonstrated loss of mutL homolog 1 expression in 1 patient and combined mutS homolog 2/mutS homolog 6 loss in 2 patients. Germline analysis in these patients identified a pathogenic *mutL homolog 1* variant in 1 and likely pathogenic *mutS homolog 2* variants in 2 patients.

Prior Korean literature comprises approximately 10 isolated MTS case reports, precluding reliable estimation of the proportion of patients meeting MTS criteria. In our cohort, 13.2% of patients with SC met clinical or molecular criteria for MTS. This finding supports the clinical relevance of MTS assessment in patients with SC, given the established association between sebaceous neoplasm and MTS or Lynch syndrome in Western cohorts.^3^^,^^5^

The proportion of extraocular SCs was significantly higher in patients with MTS than in those without MTS. This observation is consistent with prior studies suggesting that tumor location may provide a diagnostic clue.^3^

This study has several limitations, including its retrospective single-center design and the selective molecular and germline testing, which may introduce bias and limit molecular characterization of the cohort.

Collectively, this study represents the first reported detailed assessment of the proportion and clinical characteristics of MTS in Korean patients with SC. These findings highlight the association of MTS and extraocular SC, integrating clinical risk evaluation with targeted germline MMR testing.

## Conflicts of interest

None disclosed.
